# A Web-Based Psychoeducational Intervention Program for Depression and Anxiety in an Adult Community in Selangor, Malaysia: Protocol of a Randomized Controlled Trial

**DOI:** 10.2196/resprot.4622

**Published:** 2016-06-21

**Authors:** Siti Fatimah Kader Maideen, Sherina Mohd-Sidik, Lekhraj Rampal, Firdaus Mukhtar, Normala Ibrahim, Cheng-Kar Phang, Kit-Aun Tan, Rozali Ahmad

**Affiliations:** ^1^ Universiti Putra Malaysia Selangor Malaysia; ^2^ Ministry of Defence Kuala Lumpur Malaysia

**Keywords:** Web-based intervention, randomized controlled trial, depression, anxiety, psychoeducation, community, Malaysia

## Abstract

**Background:**

Mental disorders are a major public health problem and are debilitating in many nations throughout the world. Many individuals either do not or are not able to access treatment. The Internet can be a medium to convey to the community accessible evidenced-based interventions to reduce these burdens.

**Objective:**

The objective of this study is to investigate the effectiveness of 4 weeks of a Web-based psychoeducational intervention program for depressive and anxiety symptoms in the community of Selangor, Malaysia.

**Methods:**

A two-arm randomized controlled trial of a single-blind study will be conducted to meet the objective of this study. We aim to recruit 84 participants each for the intervention and control groups. The recruitment will be from participants who participated in the first phase of this research. The primary outcomes of this study are depressive and anxiety scores, which will be assessed using the Patient Health Questionnaire 9 and Generalized Anxiety Disorder 7, respectively. The secondary outcome includes mental health literacy of the participants, which will be assessed using the self-developed and adapted Mental Health Literacy Questionnaire. The psychoeducational intervention program consists of four sessions, which will be accessed each week. The depressive and anxiety symptoms will be compared between participants who participated in the psychoeducational program compared with the control group. Depressive and anxiety scores and mental health literacy will be assessed at week 1 and at follow-ups at week 5 and week 12, respectively.

**Results:**

The psychoeducational intervention program consists of four sessions, which will be accessed at each week. The depressive and anxiety symptoms will be compared between the intervention and control groups using a series of mixed ANOVAs. Depressive and anxiety scores and mental health literacy will be assessed at week 1 and at two follow-ups at week 5 and week 12, respectively.

**Conclusions:**

To our knowledge, this study will be the first randomized controlled trial of a Web-based psychoeducational intervention program for depression and anxiety in an adult community in Malaysia. The results from this study will determine the effectiveness of a psychoeducational intervention program in the management of depression and anxiety among adults in the community. If proven to be effective, the intervention can serve as a new modality to manage and reduce the burden of these disorders in the community.

**ClinicalTrial:**

International Standard Randomized Controlled Trial Number (ISRCTN): 39656144; http://www.isrctn.com/ISRCTN39656144 (Archived by WebCite at http://www.webcitation.org/6hSVhV71K)

## Introduction

Mental disorders are a major global public health problem and have a debilitating effect [1]. The burden and disability due to mental disorders are huge and yet many are still untreated and do not receive professional care [2]. Although mental disorders cause approximately 60% of unavertable burden, only a limited percentage of people receive effective treatment [3]. It is estimated that 60% of depressed individuals are not being treated [4]. This is particularly for those patients seeking cognitive behavioral therapy (CBT), which is difficult to obtain due to the insufficient mental health professionals in CBT [5] and lack of services in some geographical locations [6]. Furthermore, the stigma attached to mental health problems [7,8] and discrimination toward mental illness [7] causes many individuals to seek confidential services that assure anonymity.

Lack of psychological treatment and poor compliance with medications [8], cost of treatment, limited mobility due to illnesses [6], and lack of transportation are factors that favor the Internet as a possible medium to deliver interventions to cope with these barriers. Easy access to treatment [6] and the convenience, anonymous accessibility, and programs tailored to individuals’ needs as well as information that can be updated over time are some of the advantages of interventions delivered through the Internet as compared to the conventional method [5]. In addition to reducing the cost and time of travelling, participants are also able to access the resources at home and as often as they like [9].

Many patients report an inclination for self-help treatments. The Internet can be a medium for delivering such services and evidence shows that automated, professionally developed self-help psychological interventions can be effective [10]. Internet programs without the input of professionals can still be effective [10]. Internet interventions have been shown to reduce depression, anxiety, panic disorder, posttraumatic disorders, eating disorders, and insomnia [5]. Prevention and treatment of mental disorders, especially depression and anxiety disorders, through the Internet are increasing [10]. Several Internet-based interventions were found to reduce depression and anxiety symptoms [6,8,10-13].

A systematic review of the role of the Internet in managing depression and anxiety showed that the Internet is used as a source of information often among depressed and anxious patients [8]. Internet-based CBT programs significantly reduce the severity of generalized anxiety disorder (GAD), panic disorder, obsessive-compulsive disorder, posttraumatic stress disorder, and social anxiety disorder. The study also demonstrated that confidence of participants in managing their problems is also increased [6].

A study by Straten et al [14] of 213 participants found that self-help interventions are effective in reducing the symptoms of depression and anxiety. The study also found that those with severe baseline scores and those who completed the entire session benefit most from the program. In another study, guided self-help treatment had similar effects as face-to-face psychotherapy for the treatment of depression and anxiety [15].

A variety of psychological interventions are available for treating and managing depression and anxiety, one of them being psychoeducation. Psychoeducational interventions are educational interventions offered to individuals with psychological or physical illnesses, which are available in both active and passive forms [16].

A meta-analysis on depression, anxiety, and psychological distress showed that brief, passive psychoeducational interventions are useful to reduce depression and psychological distress [16]. Passive psychoeducational interventions are relatively cheap, easy to implement, and can be done by nonprofessionals [16]. Brief intervention programs are shown to yield positive results [17].

In another review on psychological therapies for mood disorders in adults, it was found that psychoeducation is effective in treating depression [18]. Patients with mild symptoms of depression benefit more from psychoeducation and were found to have better quality of life [19]. The study also showed that group psychoeducation is effective in the short term in reducing the scores of depression. Significant reduction in depressive symptoms through a brief mailed intervention was also demonstrated in a study by Geisner and colleagues [20]. It has also been shown that psychoeducation is effective in reducing mild-to-moderate depression [21].

A study by Mackinnon and colleagues [17] comparing the outcome of CBT and a depression information website showed a reduction in depressive symptoms in both intervention groups compared to the control group. The study also found that the effect persisted at 12 months of follow-up.

A study comparing the effectiveness of two Internet interventions found that both CBT and psychoeducation reduce depressive symptoms in the intervention group as compared to the control group [11]. The information site for depression was also found to improve the participants’ knowledge on their medical, psychological, and preference of lifestyle treatments.

Due to the importance and significance of self-management to reduce depression and anxiety, we aim to investigate the effectiveness of a 4-week Web-based psychoeducational intervention program for depressive and anxiety symptoms in the community of Selangor, Malaysia. As far as we know, there is currently no available published data on Internet-based management in Malaysia on these disorders. Therefore, this study was designed to evaluate the effectiveness and applicability of a Web-based psychoeducational intervention program in our population.

## Methods

### Study Design

A two-arm parallel randomized controlled trial (RCT) of a single-blind study will be conducted to compare 4 weeks of a Web-based psychoeducational intervention program versus a waitlist control group. This study (phase 2) is a continuation of a preliminary study (phase 1), which consisted of a cross-sectional survey to detect depression and anxiety among adults in community households in the state of Selangor, Malaysia. Selangor is one of the 13 states in Malaysia, with the highest level of urbanization. In the previous study, the cross-sectional study was conducted in three of nine districts in Selangor, namely Hulu Langat, Klang, and Sepang. The study population of this study is only representative of Selangor.

### Inclusion and Exclusion Criteria

Participants who participated in the first phase of this study will be invited to participate in this study. The inclusion criteria of this study are: (1) participants who have participated in the first phase of this study and who are still living in Selangor, (2) have access to a computer and have an Internet connection, and (3) are Internet-literate. Potential participants will be excluded if they are currently receiving any psychotherapy for diagnosed psychiatric disorders.

### Recruitment

A total of 1556 participants participated in the first phase of this study, entitled “Contributing Factors of Common Mental Health Disorders in Selangor.” Out of these, 634 participants provided complete contact information. This serves as the sampling frame in this study. A team of trained enumerators will visit the participants’ households to brief them about the study. Their personal particulars will be updated and their Internet literacy will be checked.

**Figure 1:**
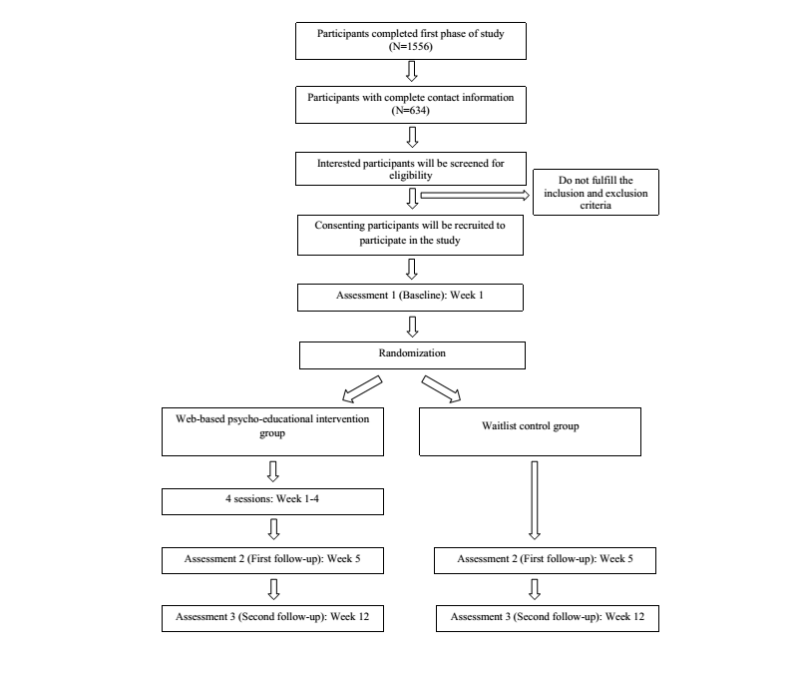
Flowchart of study design.

Eligible participants will be invited to participate in the study (second phase) through a phone call by a trained research assistant. They will be briefed about the study again. A respondent information sheet containing detailed information about the study, website log-in information, and a consent form will be emailed to the participants. After consenting to participate in the study, the participants will complete the baseline online assessment and then will be randomly allocated to either the intervention or the control group (Figure 1).

### Randomization

A list of eligible participants who consented to participate in the study will be numbered. Using a random number table, the participants will be randomly allocated to either the intervention or the control group. This study is a single-blinded trial in which the participants will not know whether they are in the intervention or the control group. However, the researcher-in-charge will be aware of the group allocation.

### Intervention

#### Psychoeducational Intervention Program (“Komuniti Sihat”)

“Komuniti Sihat,” which means “A Healthy Community,” is a brief Web-based psychoeducational intervention program. It was developed based on the findings from the first phase of this study, which explored the predictors of depression [22] and anxiety in the community of Selangor, Malaysia. The predictors of depression and anxiety were the presence of chronic diseases, serious problems at work, serious financial constraint, high perceived stress, domestic violence, low self-esteem, and unhappy relationships with children, spouse, and family. These predictors were incorporated in the program.

The intervention program consists of four sessions, with each session accessed each week. The first session includes a description of depression and anxiety, facts and myths, causes, symptoms, available treatments, self-help tips, and contact information for help. The second session involves some CBT and positive psychology. This session includes: what we need to understand, why we need to change our thinking, what we need to do, ways to think positive, ways to solve problems, relaxation techniques, and general ways to achieve good mental health. The third and fourth sessions are mainly to address stressful life events based on the findings from the first phase of the study. The third session is on techniques on how to deal with chronic diseases, stress, low self-esteem, and domestic violence, whereas the fourth session deals with issues of unhappy relationships with spouse and children, financial constraints, and problems at work.

A pilot study was carried out among 40 participants before data collection in February 2014. Errors and mistakes in the program were identified through comments obtained from the participants and were corrected accordingly. The content of this program was also reviewed by a group of expert panels, including a family medicine specialist, two psychiatrists, two clinical psychologists, a public health physician, an epidemiologist, and a biostatistician. The content of the program was revised and refined based on the comments and suggestions received from the expert panel and were also modified to meet our target population. The development of the program, process of reviewing, amendments, and completion of the program took 8 months to complete.

The four-session program is delivered online on a weekly basis. It takes approximately 15 to 20 minutes to complete each session. The participants will be taught on how to log on and use the website during the first visit to their respective households. Participants’ email addresses will be registered by an information technology technician based on the allocation group. Thereafter, the participants will log on to the website [23], using their email addresses and passwords, and complete the assessments and sessions on their own. Participants in the intervention group are allowed to view the full content on the website and use the program as frequently and as long as they want. The researchers can monitor each participant’s detailed log-in information in the back-end system.

#### Emails and Text Messages

Before commencement of the program, the participants will be notified of the dates for each session through email, which will also be provided in the website log-in information site. Participants will receive notification of the commencement of each session every Monday via email and a phone call and they will be given 7 days to complete the session. First reminders via text messages will be sent every Wednesday to alert the participants to log in and complete the session. For those who do not complete the session, a second and third reminder through phone calls will be done on Fridays and Sundays. These reminders are important to enhance the compliance of the participants to the program.

### Waitlist Control Group

Participants allocated to the control group will be blocked from the content of the intervention program. They will only be allowed to do the assessments. After the 12-week follow-up assessment, the participants in the control group will be offered to join the intervention program if they are interested and this is purely on a voluntary basis.

### Outcome Measures

#### Primary Outcome Measure

The primary outcome of this study will be the change in depressive and anxiety symptoms. This is defined by a change in the sum score on the Patient Health Questionnaire 9 (PHQ-9) and Generalized Anxiety Disorder 7 (GAD-7) between the baseline and follow-up assessments at weeks 5 and 12.

#### Secondary Outcome Measure

The secondary outcome of this study will be the change in the mental health literacy score. This is defined by a change in the total score on the Mental Health Literacy Questionnaire (MHLQ) between the baseline and follow-up assessments at weeks 5 and 12 (Table 1).

**Table 1:** Overview of measurements.

Instrument	Aim	Time of measurement		
		T_0_ (Baseline)	T_1_ (Posttest at week 5)	T_2_ (Follow-up at week 12)
Sociodemographics	Characteristics of participant	Yes		
PHQ-9	Symptoms of depression	Yes	Yes	Yes
GAD-7	Symptoms of anxiety	Yes	Yes	Yes
MHLQ	Mental health literacy	Yes	Yes	Yes

### Instruments

#### Patient Health Questionnaire 9

The PHQ-9 is used to measure the severity of depression based on the *Diagnostic and Statistical Manual of Mental Disorders* (Fourth Edition; *DSM-IV*) criteria. It consists of nine items, each item rated on a scale from 0 to 3, and a total score range from 0 to 27. Both the validated English [24] and Malay [25] versions of the questionnaire are used in this study.

#### Generalized Anxiety Disorder 7

The GAD-7 is used to measure the severity of anxiety based on the *DSM-IV* criteria. The seven items are each scored from 0 to 3, with an overall range score of 0 to 21. Both the validated English [26] and Malay [27] versions of the questionnaire are used in the study.

#### Mental Health Literacy Questionnaire

Some studies uses case vignettes to measure the knowledge of participants on mental health illness, such as depression, anxiety, and schizophrenia. However, the education level of a community in a developing country such as Malaysia is lower compared to developed countries. Because of this, we are unable to use case vignettes and specific facts to assess the mental health knowledge of the participants. Therefore, we had to create items measuring knowledge based on a local context, taking into account cultural-specific issues.

To assess the knowledge on depression and anxiety, eight items were developed based on the content of the psychoeducational intervention program (general knowledge on mental health). Another four items were adapted from the MHLQ from the domain of knowledge and capability [28] and were modified based on the suitability of the items measuring depression and anxiety in this study. In total, 12 items were used to assess the participant’s knowledge on depression and anxiety. The items are coded as yes and no. The items will be summed, with a total score ranging from 0 to 12. Both English and Malay versions of the questionnaire are used in this study.

### Sample Size

The sample size for this study was calculated using a formula by Lemeshow et al [29]: n=(2σ^2^[Z_1−α/2_+Z_1−β_]^2^)/([μ_1_−μ_2_]^2^).

Using a power of 80%, a confidence interval of 95%, a pooled standard deviation of 9.865, and a baseline and posttest mean score of 21.1 and 16.2, respectively, in the intervention group [11], the calculated sample size was estimated to be 64 participants. Taking into consideration a 30% dropout rate [30], the final sample size calculated was 84 participants in each group.

### Statistical Analysis

Data will be analyzed using IBM SPSS version 21.0 software. Differences between sociodemographic characteristics for the intervention and control groups will be tested using chi-square and *t* tests. A mixed between-within subjects ANOVA will be employed to compare the mean difference of the psychoeducation intervention program between the two groups at pretest, posttest (week 5), and at 2-months of follow-up (week 12). The magnitude effect size of the intervention program will be calculated using Cohen’s formula. Analyses will be conducted based on the intention-to-treat principle and for all completers.

### Trial

The trial will be conducted using the Internet as a medium of implementation of the psychoeducation intervention program. The planning of the study started on March 1, 2013, and data collection is expected to be complete on August 14, 2014. At the point of submitting this paper for publication, data analysis had not yet been completed.

### Ethics Approval and Registration

This study was approved by the University Research Ethics Committee of Universiti Putra Malaysia on August 14, 2013 (Reference No: UPM/TNCPI/RMC/1.4.18.1 JKEUPM). The study is registered in the Malaysian National Medical Research Registry (NMRR-14-698-21864) and in the ISRCTN registry (ISRCTN39656144). Consent will be obtained from all the participants who are willing to participate in the study.

## Results

By the time the manuscript was submitted to the journal, data collection was completed. The results are expected to be published in late 2015 or early 2016.

## Discussion

This RCT study protocol is aimed to investigate the effectiveness of a Web-based psychoeducational intervention for depressive and anxiety symptoms for the adult community in Selangor, Malaysia. Self-management through the Internet offers the opportunity to reach and treat the community who may suffer from mild-to-moderate depression and anxiety, and for those who are reluctant to seek for assistance from medical professionals. In addition, it can serve as an appealing modality for quick help to manage these problems. Web-based programs can provide the advantage of disclosing certain information better than face-to-face interviews. Moreover, information can be accessed 24/7 and is available to a wider community. This can be very useful when an immediate need is required for information on management.

To our knowledge, Internet-based management for both depression and anxiety in the community has not been studied in Malaysia. This study could give an insight on the effectiveness and applicability of a Web-based psychoeducation program in the current population. The strength of this study is that the psychoeducational intervention program is developed based on the predictors of depression [22] and anxiety in our own population. The program aims to address the predictors that contribute to depression and anxiety in our community. This brief Web-based program will enable the community to use easy, user-friendly, valid, and reliable tools for assessing their mental health status.

Second, the availability of the program in both English and Malay languages is an additional plus point. Participants can choose their preference language to complete the sessions. Third, the assessments are available in both languages and have been validated in our population. Fourth, the intervention program was designed to be as brief as possible to increase the acceptability of the program versus other lengthier interventions, which could further increase the dropout rates.

Nevertheless, there are some limitations in this study. One potential limitation of this study is the nature of a Web-based program itself. The Web-based program will be restricted only to those who are literate and who have access to the Internet. Although its use will be limited to people who are computer literate and who have access to the Internet, this population is increasing significantly in Malaysia. Therefore, a great number of people will be able to access this Web-based assessment and intervention program, and use it for assessment of their own mental health and seek help when required. A second limitation is assurance cannot be made that the selected participants will complete the study by themselves. However, by having a specific username and password to log in to the system, this problem would be minimized. Another potential limitation is the short duration of follow-up to assess the effect of the intervention program. However, studies have shown that effectiveness can be shown even with shorter durations [19].

The Web-based psychoeducation program could serve as a new modality to manage mild-to-moderate depression and anxiety. Our study aims to provide better recognition and management of depressive and anxiety symptoms. It also aims to educate and create awareness about depression and anxiety in the community. Depression and anxiety are the most common mental health disorders. There is a need to develop simple, brief, and effective interventions tailored to the needs of the community to reduce the burden of these disorders.

## Abbreviations

CBT: cognitive behavioral therapy
DSM-IV: Diagnostic and Statistical Manual of Mental Disorders (Fourth Edition)
GAD: generalized anxiety disorder
GAD-7: Generalized Anxiety Disorder 7
MHLQ: Mental Health Literacy Questionnaire
PHQ-9: Patient Health Questionnaire 9
RCT: randomized controlled trial
